# Immunological Dynamics and Outcomes of ABO‐Incompatible Kidney Transplants: Insights From a 5+‐Year Follow‐Up at a Transplant‐Heavy Tertiary Center in Saudi Arabia

**DOI:** 10.1155/joot/6265955

**Published:** 2026-07-03

**Authors:** Bilal Mohsin, Najla Zabani, Nasser Odah, Naief Alhowaiti, Fatmah Yamani, Afnan Al Mutairi, Lama Hefni, Muhammad Talha Kausar, Nadeem Shafique Butt, Wael Habhab

**Affiliations:** ^1^ Department of Nephrology, King Faisal Specialist Hospital & Research Center, Jeddah, Saudi Arabia, kfshrc.edu.sa; ^2^ University of Health Sciences, Lahore, Pakistan, uhs.edu.kh; ^3^ Department of Family and Community Medicine, King Abdulaziz University, Jeddah, Saudi Arabia, kau.edu.sa

**Keywords:** ABOi kidney transplant, antibody-mediated rejection, desensitization, graft rejection, immunological risk factors

## Abstract

**Background:**

ABO‐incompatible (ABOi) kidney transplantation is an essential strategy to expand the donor pool for patients with end‐stage kidney disease (ESKD). However, it poses significant immunological challenges because of the risk of graft rejection. In Saudi Arabia, where ABOi transplants are limited, there is a paucity of long‐term locoregional data on the impact of immunological factors on graft outcomes. This study evaluated ABOi transplant outcomes in patients with more than 5 years of follow‐up, with a primary focus on immunological factors and their role in graft survival and rejection.

**Methods:**

We analyzed data from 24 adult patients who underwent ABOi kidney transplantation between October 31, 2015, and December 30, 2019, with follow‐up until December 31, 2024. Key immunological factors assessed included donor–recipient blood group mismatch, human leukocyte antigen (HLA) mismatches, preformed donor‐specific antibodies (DSAs) and non‐DSA anti‐HLA antibodies, flow cross‐match results, and anti‐ABO titers before and after transplantation. Posttransplant infections were also evaluated for their potential immunological impact. The desensitization protocol comprised rituximab, plasma exchange, and intravenous immunoglobulin (IVIg), with pretransplant anti‐ABO titers required to be ≤ 8. Exclusion criteria included pediatric ABOi kidney transplants and patients experiencing major vascular or surgical complications necessitating re‐exploration within 7 days posttransplant. Immunosuppressive therapy included methylprednisolone and ATG induction, with prednisolone, tacrolimus, and mycophenolate mofetil for maintenance. The studied outcomes were graft survival, rejection episodes, graft failure, and all‐cause mortality.

**Results:**

Over a mean follow‐up of 68 ± 12.6 months, graft survival was 91.7% (*n* = 22). Graft rejection occurred in 16.7% (*n* = 4) of the patients, with two cases (8.3%) of graft failure attributed to severe antibody‐mediated rejection (ABMR). Key immunological risk factors for rejection included the presence of non‐DSA anti‐HLA antibodies, low‐titer positive cross‐match, > 8 HLA mismatches, and unrelated donors. A total of 49 infectious episodes occurred in 19 recipients, of which 29 (59%) occurred within the first posttransplant year and were not associated with graft dysfunction or rejection. Posttransplant serum creatinine remained stable during follow‐up, with median values of 89.0, 88.0, 85.0, and 88.0 μmol/L at 1, 12, 36, and 60 months, respectively, and 77.0 μmol/L at last follow‐up (minimum follow‐up, 64 months). No mortality was observed.

**Conclusion:**

ABOi kidney transplantation demonstrates excellent long‐term outcomes; however, immunological factors play a pivotal role in determining the risk of rejection. Careful patient selection is warranted, as even seemingly minor immunological factors can provoke graft rejection in the context of ABO incompatibility. Posttransplant infections were not significantly associated with graft dysfunction or rejection. A small sample size allows us to report these findings; however, to review their significance, a larger cohort of patients, incorporating protocol biopsies, in a prospective multicenter study is needed to validate these findings.

## 1. Introduction

Chronic kidney disease (CKD) represents a substantial global health challenge, and end‐stage kidney disease (ESKD) necessitates kidney replacement therapy to sustain life. Among the therapeutic options, kidney transplantation stands as the gold standard, offering superior survival rates and enhanced quality of life compared to dialysis [1]. However, the escalating prevalence of CKD has dramatically widened the gap between the demand for and the availability of compatible donor organs, leading to prolonged waiting times and increased patient morbidity and mortality. To address this critical organ shortage, ABO‐incompatible (ABOi) living donor kidney transplantation has emerged as a promising strategy, expanding the donor pool and providing a timely alternative for patients lacking ABO‐compatible donors [2]. Moreover, ABOi kidney transplantation is a suitable option for patients who do not have a suitable blood group‐compatible donor, with outcomes better than remaining on hemodialysis or receiving a deceased donor transplant [3].

Although ABOi transplantation offers potential benefits, it introduces unique immunological challenges that necessitate careful consideration. These challenges include the implementation of desensitization protocols to lower ABO iso‐titers, which can lead to an increased incidence of infectious complications, bleeding risks, increased hospital stay, and higher treatment costs [4, 5].

In addition to the immediate threat of hyperacute rejection, ABO‐incompatible transplantation carries a heightened risk of antibody‐mediated rejection (ABMR), which can occur acutely or chronically [6, 7]. ABMR is characterized by the interaction of donor‐specific antibodies (DSAs) and blood‐group antibodies with endothelial cells in the graft, leading to complement activation, inflammation, and graft injury [7, 8]. The presence of concomitant human leukocyte antigen (HLA) incompatibility may further increase immunological risk by facilitating the development of de novo DSAs [7, 9]. These antibodies can target the graft endothelium, causing microvascular inflammation and ultimately contributing to graft dysfunction and failure [8, 9].

The monitoring of DSA levels and the implementation of appropriate immunosuppressive regimens are crucial for preventing and managing ABMR [10, 11].

In addition to anti‐ABO antibody burden, other immunological factors may influence outcomes in ABO‐incompatible kidney transplantation, including donor‐specific anti‐HLA antibodies, overall HLA incompatibility, and baseline sensitization status. These factors may modify the risk of ABMR and contribute to subsequent graft dysfunction or graft loss [12, 13]. Posttransplant infections, which are more frequent in ABOi recipients due to intense immunosuppression, can indirectly influence immunological outcomes. Infections can trigger an inflammatory cascade, potentially leading to increased DSA production and exacerbating rejection risks. For example, cytomegalovirus (CMV) has been implicated in the development of chronic allograft nephropathy and increased rates of rejection [14, 15]. Therefore, careful management of infections is essential not only for preventing direct morbidity but also for mitigating their potential impact on graft survival [16, 17].

Even in the face of these concerns, ABOi kidney transplant recipients have better clinical outcomes than ESKD patients on the waiting list for kidney transplantation [7].

ABOi kidney transplantation, while expanding the donor pool, poses a significant immunological challenge. The intricate interplay between preformed antibodies, HLA mismatches, non‐DSA antibodies, and posttransplant infections underscores the need for a comprehensive understanding of the pathophysiological mechanisms involved.

This study evaluated ABOi transplant outcomes in patients with at least 5 years of follow‐up at a kidney transplant center in Saudi Arabia. This research seeks to provide insights into the immunological behavior of the local population regarding ABOi kidney transplantation, which is needed because of the paucity of local data.

## 2. Materials and Methods

### 2.1. Study Design and Population

We included 24 adult ABOi kidney transplant recipients who underwent kidney transplantation between October 31, 2015, and December 30, 2019. Chart reviews of these patients were conducted until December 31, 2024. Pre‐ and posttransplant data from all patients were retrospectively analyzed. The exclusion criteria were as follows: pediatric ABOi kidney transplantation, ABOi kidney transplantation with significant surgical or vascular complications, surgical exploration within 7 days, and failure to complete follow‐up for at least 5 years.

This study was approved by the Institutional Review Board (IRB) of the hospital’s Research Ethics Committee (IRB number 2020‐48). Each patient’s file was assigned a study code to facilitate data collection and electronic data entry. Only the principal investigator and actively associated members of the study team had access to patients’ personal identifiers.

### 2.2. Data Collection

Using the Statistical Package for the Social Sciences software and an MS Excel sheet, we extracted relevant data from the medical records of patients who underwent kidney transplantation at our center during the specified period. Demographic characteristics, clinical comorbidities, and immunological parameters, including donor–recipient blood group, sex comparison, HLA mismatch, performed DSA, flow cross‐match, desensitization protocol, renal profile, patient and transplant outcomes, and infectious and noninfectious complications, were obtained.

Graft loss was defined as retransplantation, dialysis, or death with a functional graft. Graft rejection was defined as histologically evident rejection based on the Banff criteria [18], confirmed by an ultrasound‐guided transplant kidney biopsy. Infections were identified using recorded positive blood and urine cultures, serology, and polymerase chain reaction (PCR).

### 2.3. Histological Assessment

We performed a renal biopsy on each postkidney transplant patient who showed no improvement in serum creatinine levels in the absence of known surgical problems within the first 2 weeks after the transplant. In subsequent follow‐ups, every patient with a serum creatinine increase of > 27 μmol/L in the absence of dehydration, high Prograf level, bladder outflow blockage, or active infection underwent ultrasound‐guided transplant kidney biopsy. Additionally, biopsies were performed in patients with new‐onset proteinuria and hematuria.

We did not perform protocol biopsies following kidney transplantation because the patient denied consent.

### 2.4. Desensitization Protocol for ABOi Kidney Transplantation

All ABOi kidney graft patients were desensitized before transplantation. The goal of this phase was to reduce ABO antibodies (isoagglutinins) to a target titer of ≤ 8 in the peri‐OR interval using rituximab, plasma exchange, and immunoglobulins. The desensitization protocol consisted of the following steps:

1. All kidney transplant recipients received rituximab (375 mg/m^2^ BSA; approximate dose, 500 mg) 2–3 weeks before the initiation of desensitization.
2. Plasma exchange was conducted based on the isoagglutinin titer prior to transplantation.
(1) PE volumeL=1−HCT×body weightkg×0.07.
 Human albumin 5% was used as the replacement fluid. Fresh frozen plasma (FFP) from the donor group was required before a few sessions of surgery. Serum fibrinogen and partial thromboplastin time (PTT) were regularly evaluated, and low fibrinogen levels were addressed with donor blood group FFP given during the next session of plasma exchange. Post‐OR plasma exchange was continued for three or more sessions until the anti‐ABO antibody titer stabilized at 16 or below for 48 h.
3. After each plasma exchange, intravenous immunoglobulin (IVIg; 0.2 g/kg) was administered at a cumulative dose of 1–2 g/kg.
4. The transplant was usually conducted at an antibody titer of ≤ 8; however, it was occasionally conducted at a titer of 16.
5. Anti‐ABO isoagglutinin titers were measured using the gel column agglutination technique with manual visual interpretation, consistent with standard transfusion laboratory practice.

#### 2.4.1. Immunological Risk Assessment:

A low‐titer positive cross‐match was defined as a mean channel shift < 150 for both T‐ and B‐cell flow cross‐match and DSA mean fluorescence intensity < 1000, thresholds commonly applied in low‐risk immunological profiles in ABO‐incompatible transplantation (ABOi kidney transplant phase).

### 2.5. HLA Antibody Testing and Immunological Risk Assessment

HLA antibody screening was routinely performed as part of the pretransplant immunological assessment after identification of a potential donor and during donor–recipient compatibility evaluation.

HLA antibody testing was performed using a single‐antigen bead assay. Donor and recipient HLA typing included class I loci HLA‐A, HLA‐B, and HLA‐C and Class II loci HLA‐DRB1, HLA‐DQA1, HLA‐DQB1, HLA‐DPA1, and HLA‐DPB1. Genomic DNA was extracted using the QIA Symphony platform (Qiagen), and HLA typing was performed primarily by next‐generation sequencing (NGS), with reverse sequence‐specific oligonucleotide probing (RSSO) used where applicable.

HLA mismatch burden was assessed by direct comparison of donor and recipient HLA typing results across the eight evaluated HLA loci. Because each locus contains two alleles, each locus could contribute 0, 1, or 2 donor–recipient allele‐level mismatches. Therefore, the cumulative HLA mismatch score was expressed as the total number of allele‐level mismatches across these eight loci, ranging from 0/16, indicating complete allelic matching, to 16/16, indicating complete allelic mismatching.

DSA assessment was performed 4 weeks prior to initiation of the desensitization protocol in order to avoid false‐positive cross‐match due to Rituximab.

Panel reactive antibody (PRA)/calculated panel reactive antibody (cPRA) values were not uniformly available for all recipients; sensitization status was characterized using HLA antibody results, cross‐match findings, and donor–recipient HLA mismatch rather than percentage PRA/cPRA values.

### 2.6. Induction and Maintenance Immunosuppressive Regime

All transplant recipients received a consistent immunosuppressive regimen in accordance with the protocol of our center. Thymoglobulin was administered intravenously for induction at a total dose of 4.5–6 mg/kg to all 24 patients, with the first dose given intraoperatively at 1.5 mg/kg. Mycophenolate mofetil was started on Day 0, while tacrolimus was started on Days 2‐3 after kidney transplantation once the serum creatinine levels dropped to 50% of the pretransplant values. Methylprednisolone was administered in doses of 250 mg IV on Day 0, 125 mg on Day 1, and 1 mg per kilogram on Day 2, with a taper of 5 mg every day until a daily dose of 20 mg was reached. Subsequently, it was lowered by 5 mg per week until a maintenance dose of 5 mg per day was reached.

Tacrolimus trough levels were maintained at 8.5–10 ng/mL for the first 3 months, and then steadily decreased to 7.5–8.5 ng/mL by 6 months, 6–7.5 ng/mL by the end of 1 year, and finally between 4.5 and 6 ng/mL. The target level was slightly higher for patients treated for acute rejection. When tacrolimus toxicity was detected, the dose was lowered, and everolimus was introduced instead. When tacrolimus was combined with everolimus, the target levels were 3.5–4.5 ng/mL and 4.5–5.5 ng/mL, respectively. Most patients received MMF twice daily at a dose of 1000 mg. This was reduced to 750 mg twice daily if the body surface area was < 1.2 m^2^ or the patient weighed < 50 kg.

### 2.7. Surgical Anastomosis Time

The anastomosis time varied between 30 and 45 min, depending on the number of vessels.

### 2.8. Graft Rejection

All rejections were biopsy‐confirmed and classified according to the Banff classification [18] and treated with 500 mg IV methylprednisolone daily for 3 days. Following ABMR, plasma exchange with IVIg administration was performed daily or on alternate days. Following TCMR, patients were treated with 500 mg IV methylprednisolone daily for three days, with steroid tapering based on clinical response. Thymoglobulin was considered for patients with Banff categorization 2a or higher.

### 2.9. Prophylaxis

All D+/R+ patients received valganciclovir (450 mg daily) for 90 days to prevent CMV infection; D+/R− patients received the same prophylaxis for six months.

Cotrimoxazole (80 mg), administered orally daily, is recommended as a PJP prophylaxis for 6–9 months. Valganciclovir and cotrimoxazole were administered once the GFR exceeded 30 mL/min/1.73 m2 BSA.

### 2.10. Outcomes

The main outcomes were graft survival, graft rejection, graft failure, and all‐cause mortality. The studied infections included urinary tract infections (UTIs), viral infections (COVID‐19, Influenza, BK virus, and CMV), and systemic fungal infections as documented by positive cultures or PCR.

## 3. Results

A total of 39 patients underwent ABOi kidney transplant between October 31, 2015, and December 30, 2019, at our center. A total of *n* = 24 patients met the study criteria and were followed up until December 31, 2024. The patients’ demographic and clinical characteristics are shown in Table 1.

**TABLE 1:** Demographic and clinical characteristics of ABO‐incompatible kidney transplant patients (*n* = 24).

Variable	*n* (%)	Mean ± SD/median (IQR)
Donor		
Age (years)		32.7 ± 7.5
Gender		
Male	10 (41.7)	
Female	14 (58.3)	
BMI		24.1 ± 5.7
Recipient		
Age (years)		44.2 ± 13.5
Gender		
Male	11 (45.8)	
Female	13 (54.2)	
BMI		26.0 ± 5.5
Blood group combination		
A+ ⟶ B+	1 (4.2)	
A+ ⟶ O+	15 (62.5)	
A+ ⟶ O−	1 (4.2)	
AB+ ⟶ A+	2 (8.3)	
B+ ⟶ A+	3 (12.5)	
B+ ⟶ O+	2 (8.3)	
Comorbidities		
Diabetes	9 (37.5)	
Hypertension	20 (83.3)	
Cardiac disease	1 (4.2)	
COPD	0 (0.0)	
Malignancy	0 (0.0)	
HIV/AIDS, autoimmune disease	0 (0.0)	
Chronic HCV infection	1 (4.2)	
HLA cross‐match		
Negative	22 (91.6)	
Negative, positive for B cell	1 (4.2)	
Negative with DSA	1 (4.2)	

*Note:* Mean ± SD or median (IQR) reported according to distribution of the variable data.

Abbreviations: COPD, chronic obstructive pulmonary disease; DSA, donor‐specific antibodies; WBC, white blood cells.

The majority of blood group combinations among the transplant pairs were A+ to O+ (62.5%). HLA cross‐matching was negative in 22 (91.6%) recipients. On the day of surgery, 91.6% (*n* = 22) of recipients had an isoagglutinin titer of ≤ 8 or less, while two had a titer of 16 (Table 2).

**TABLE 2:** Isoagglutinin titers at time of desensitization, operation, Day 1 postop and discharge.

Isoagglutinin titers	Desensitization	Operation	Day 1 postop	Discharge
1	0 (0.0)	0 (0.0)	1 (4.2)	3 (12.5)
2	0 (0.0)	4 (16.7)	2 (8.3)	3 (12.5)
4	2 (8.3)	7 (29.2)	11 (45.8)	10 (41.2)
8	2 (8.3)	11 (45.8)	9 (37.5)	3 (12.5)
16	8 (33.3)	2 (8.3)	1 (4.2)	0 (0.0)
28	0 (0.0)	0 (0.0)	0 (0.0)	1 (4.2)
32	9 (37.5)	0 (0.0)	0 (0.0)	2 (8.3)
64	3 (12.5)	0 (0.0)	0 (0.0)	2 (8.3)

*Note:* Data are expressed as *n* (%).

Graft survival was 91.7% (*n* = 22) over a mean follow‐up duration of 64 ± 12.6 months. Four patients experienced graft rejection (16.7%), all within the first 2 months of kidney transplantation. Of these patients, three had ABMR, and one had TCMR. Of the patients who had ABMR, two had graft failure (8.3%) within 3 weeks of transplantation. Two patients recovered with treatment and had functioning grafts at the date of their last follow‐up (64 months). Table 3 presents the characteristics of patients with graft recovery and graft failure after rejection.

**TABLE 3:** Characteristics of patients with graft rejection with graft failure/recovery.

Characteristic	Graft failure (2 cases)		Graft recovery (2 cases)	
Recipient age	48 years	48 years	40 years	21 years
Donor age	45 years	42 years	27 years	43 years
Date of transplant	11‐May‐2018	11‐Dec‐2015	15‐Sep‐2019	11‐Nov‐2019
Date of graft rejection	14‐May‐2018	21‐Dec‐2015	25‐Sep‐2019	15‐Dec‐2019
Donor relation	Unrelated wife	Unrelated wife	Cousin	Mother
Gender association	F to M	F to M	M to M	F to F
Type of rejection	Hyperacute severe ABMR	Cortical necrosis due to severe acute ABMR	Acute ABMR	TCMR with C4d positive
Banff classification	ACUTE ABMR; C4d+; severe PTC; acute tubular injury	ACUTE ABMR; C4d+; severe PTC; glomerulits	ACUTE ABMR; C4d+; PTC; acute tubular injury	Acute TCMR Grade 1 B with C4d positive with PTC
Final cross‐match	Negative with DSA to DQ 778 MFI and presence of multiple non‐DSA anti‐HLA antibodies	Positive B cell with low titers	Positive B and T cell with low titers	Negative
HLA mismatch^∗^	10:16	9:16	13:16	1:16
Blood group (recipient)	O+	O+	O+	O+
Blood group (donor)	A+	A+	B+	B+
ABO isotiter prior to desensitization	16	64	4	16
ABO isotiter prior to operation	8	8	8	16
ABO isotiter D1	4	8	4	2
ABO isotiter prior to discharge	32	4	1	28
No. of plasma exchange pretransplant	3	3	2	3
No. of plasma exchange posttransplant	10	6	9	9

^∗^HLA mismatch is expressed as the number of donor–recipient mismatched loci out of 16 evaluated loci; thus, 10:16 indicates 10 mismatches across 16 tested loci, whereas 1:16 indicates only 1 mismatch.

Patients experiencing graft rejection leading to failure demonstrated either a positive cross‐match with low titers or a negative cross‐match with low‐titer DSA to DQ and multiple non‐DSA anti‐HLA antibodies. In these cases, the HLA mismatch loci were more than 8 out of 16, and the donor–recipient sex combination was female–male. The donors were unrelated wives, and the donor–recipient blood group was A+ to O+. These patients underwent three sessions of plasma exchange pretransplant with an ABO isotiter of 8 or less on Day 1 postkidney transplant. Histopathology confirmed ABMR, with evidence of hyperacute ABMR and cortical necrosis. There were no instances of intraoperative hypotension, blood transfusions, or postoperative vascular or surgical complications in these patients.

Patients who experienced rejection but achieved recovery had acute ABMR and TCMR with C4d positivity, respectively. The donors were a cousin and the mother, respectively. The patient with acute ABMR had a higher number of HLA mismatches and a positive cross‐match with low titers, whereas the patient with TCMR had a lower number of HLA mismatches and a negative flow cross‐match but was suspected of noncompliance with medications. The donor–recipient blood group was B+ to O+, with an ABO isotiter of 4 on Day 1 postkidney transplant.

Both patients had functional grafts with a follow‐up duration of > 55 months.

A total of 49 infectious episodes were recorded in 19 patients (79.2%); 5 patients had infection‐free follow‐up of more than 5 years. UTIs were most common (23/49), followed by COVID‐19 (11/49) and Influenza A (7/49). No episode of CMV, BK, or fungal infection was observed. Infection incidence was highest in females (52.6%), diabetics (47.4%), and patients with prior rejection episodes (10.5%). There was no statistical association between infections and rejection.

The association of ABO isoagglutinin titers, infections, donor–recipient blood group, age, and sex did not reach statistical significance. Donor age and HLA mismatch showed a statistical association with graft rejection (Tables 4 and 5).

**TABLE 4:** Association of high isoagglutinin titers and blood group of donors and recipients with graft rejection in ABO incompatible kidney transplant patients.

Variable	Graft rejection	Graft rejection	*p* value^1^
	Yes (*n* = 4)	No (*n* = 20)	
High isoagglutinin titers^2^ at time of desensitization	3 (75.0)	17 (85.0)	0.5440
High isoagglutinin titers^2^ at time of operation	1 (25.0)	3 (15.0)	0.5440
High isoagglutinin titers^2^ on Day 1 postop	0 (0.0)	1 (5.0)	1.0000
High isoagglutinin titers^2^ at time of discharge	2 (50.0)	3 (15.0)	0.1793
Donor blood type			
A+	2 (50.0)	15 (75.0)	
AB+	0 (0.0)	2 (10.0)	0.4561
B+	2 (50.0)	3 (15.0)	
Recipient blood type			
A+	0 (0.0)	5 (25.0)	
B+	0 (0.0)	1 (5.0)	
O+	4 (100.0)	13 (65.0)	0.6800
O−	0 (0.)	1 (5.0)	

^1^Fisher’s exact test.

^2^Isoagglutinin titers were categorized as high above 8.

**TABLE 5:** Factors influencing graft rejection in transplant recipients: a comparative analysis.

		Graft rejection			*p*
		Yes	No	Total	
Total *n* (%)		4 (16.7)	20 (83.3)	24	

Gender donor	F	3 (75.0)	11 (55.0)	14 (58.3)	0.615
	M	1 (25.0)	9 (45.0)	10 (41.7)	

Gender recipient	F	1 (25.0)	12 (60.0)	13 (54.2)	0.300
	M	3 (75.0)	8 (40.0)	11 (45.8)	

Gender association donor to recipient	F‐F	1 (25.0)	8 (40.0)	9 (37.5)	0.584
	F‐M	2 (50.0)	3 (15.0)	5 (20.8)	
	M‐F	0 (0.0)	4 (20.0)	4 (16.7)	
	M‐M	1 (25.0)	5 (25.0)	6 (25.0)	

Recipient age	Median (IQR)	44.0 (35.2–48.0)	49.0 (36.2–55.0)	47.0 (36.2–53.5)	0.352

Donor age	Median (IQR)	42.5 (38.2–43.5)	32.5 (25.0–37.0)	33.0 (25.8–40.0)	0.033

Infection	No	1 (25.0)	4 (20.0)	5 (20.8)	1.000
	Yes	3 (75.0)	16 (80.0)	19 (79.2)	

HLA mismatch	≤ 8:16	1 (25.0)	18 (90.0)	19 (79.2)	0.018
	> 8:16	3 (75.0)	2 (10.0)	5 (20.8)	

*Note:* HLA mismatch is expressed as the number of donor–recipient mismatched loci out of 16 evaluated loci; thus, e.g., 10:16 indicates 10 mismatches across 16 tested loci, whereas 1:16 indicates only 1 mismatch.

Table 6 shows the trend of serum creatinine over the follow‐up duration of the study. Posttransplant serum creatinine remained generally stable over time. Median serum creatinine was 89.0 μmol/L (IQR, 73.5–107.5) at 1 month, 88.0 μmol/L (72.0–108.0) at 12 months, 85.0 μmol/L (73.0–106.5) at 36 months, and 88.0 μmol/L (64.5–100.0) at 60 months. At the last follow‐up, after a minimum follow‐up of 64 months, the median serum creatinine was 77.0 μmol/L (54.9–99.1).

**TABLE 6:** Serum creatinine as median IQR (posttransplant, µmol/L).

1 month	89.0 (73.5–107.5)
12 months	88.0 (72.0–108.0)
36 months	85.0 (73.0–106.5)
60 months	88.0 (64.5–100.0)
Last follow‐up^∗^ (64 months min.)	77.0 (54.9–99.1)

*Note:* Data are presented as median (interquartile range [IQR]) in μmol/L.

^∗^“Last follow‐up” refers to the most recent available serum creatinine measurement, with a minimum follow‐up duration of 64 months.

**FIGURE 1:**
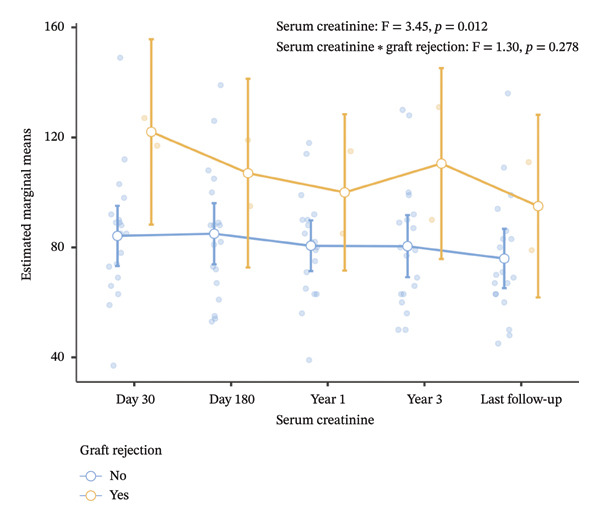
Longitudinal analysis of serum creatinine levels in patients by graft rejection status.

Figure 1 shows that serum creatinine levels change over time in both groups. In general, patients with graft rejection (orange line) have higher serum creatinine levels than those without graft rejection (blue line), although the difference between the groups varies at different time points.

The results of the repeated‐measures ANOVA indicated that changes in serum creatinine levels with the duration of follow‐up were statistically significant (*p* = 0.012); however, the interaction between time and graft rejection status was not (*p* = 0.278). This implies that although serum creatinine levels are affected by time, the pattern of change over time does not differ significantly between those with and without graft rejection.

By the last follow‐up, serum creatinine levels in the graft rejection group appeared to decrease toward the levels observed in the nonrejection group.

## 4. Discussion

This is the first loco‐regional study conducted at a transplant‐heavy tertiary care center in the southern region of Saudi Arabia, reviewing immunological factors and their association with graft outcomes. Our study notes a complex interplay of immunological factors that, when carefully reviewed, reveals interesting findings.

We observed that a high ABOi isotiter prior to desensitization can be a contributing factor for graft rejection and failure. We also observed that in the presence of an immunological burden of ABOI kidney transplant, otherwise weak immunological factors can contribute to the precipitation of graft rejection and occasionally graft failure. These include unrelated donor–recipient relationships, low‐titer non‐donor‐specific anti‐HLA antibodies (non‐DSA), weakly positive flow cross‐match to B cells, and HLA mismatch. We also noted that recipients who developed graft dysfunction or rejection more frequently exhibited a concomitant presence of one or more of these immunological factors, although no causal relationship can be inferred. In contrast, we did not find any significant association between other factors, including recipient age, donor–recipient sex disparity, clinical comorbidities, episodes of bacterial or viral infections, and graft dysfunction. This observation suggests the likelihood of immunological factors as the drivers of graft rejection in ABOi kidney transplantation, a finding that has been marginally reviewed by locoregional studies [19, 20].

High isohemagglutinin titers, which directly target the donor’s blood group antigens, are well‐documented culprits in hyperacute rejection. In our study, the amplification of graft rejection risk in the presence of unrelated donor–recipient relationships adds another dimension to risk assessment. Research suggests that genetic mismatches in unrelated donors may result in increased immunogenicity, intensifying the recipient’s immune response against the graft [21]. Non‐DSA, although generally considered less likely than DSAs to directly mediate rejection or graft failure, may still exert additive or synergistic effects when present alongside other immunological risk factors, as suggested by both our findings and prior literature [22]. International studies support our findings that weakly positive flow cross‐match to B cells indicates a subtle yet noteworthy humoral response that can further destabilize graft integrity [23]. Donor–recipient HLA mismatch emerged as an important immunological factor associated with graft dysfunction in ABO‐incompatible kidney transplantation. In our cohort, a greater mismatch burden across the typed HLA loci appeared to be associated with less favorable graft outcomes. This observation is consistent with prior literature suggesting that increased HLA incompatibility may contribute to immunological injury and adverse graft outcomes in kidney transplantation, including ABO‐incompatible settings [24, 25], with a mention to try to minimize the mismatch as much as possible, especially for Class II MHC antigens [26].

In contrast, factors such as age and sex disparity did not show a significant influence on graft outcomes in our study. Although previous research has hypothesized potential correlations, such as hormonal influences or differences in immune reactivity, our findings suggest that these factors play a minimal role in ABOi transplantation under controlled desensitization conditions [27].

The findings of this study have important implications for clinical practice. Identifying recipients at a heightened risk due to the interplay of multiple minor immunological factors can guide pretransplant counseling and posttransplant care. The development of risk scores tailored to the local population, incorporating these elements, could help with patient selection, risk stratification, enhance predictive accuracy, and tailor immunosuppressive regimens more effectively. Moreover, the lack of association with nonimmunological factors reinforces the need to prioritize immunological evaluation in ABOi transplantation protocols.

We observed that the nuanced interplay of immunological factors in ABOi kidney transplantation underscores the importance of individualized approaches to transplantation. Although advancements in desensitization have significantly improved success rates, the findings of this study advocate for heightened vigilance in cases presenting with multiple minor immunological risk factors. Future research should aim to elucidate the underlying mechanisms of these synergistic effects and explore strategies to mitigate their impact.

## 5. Limitations

The small sample size (*n* = 24) does not prevent us from gaining insights into subtle associations between various immunological factors and graft outcomes; however, it restricts the statistical power to commit significant associations between immunological factors and graft outcomes. We did not have the opportunity to perform protocol biopsies due to a lack of patient consent, which might have underdiagnosed subclinical graft rejection. Standardized numeric PRA/cPRA values were not consistently available for all patients; therefore, baseline sensitization could not be uniformly reported using percentage PRA/cPRA and was instead described using HLA antibody testing and cross‐match‐based immunological assessment. As a single‐center study, the results may not be generalizable to diverse populations with varying immunosuppressive practices. Finally, retrospective data collection introduces potential selection bias and the possibility of overlooking important clinical events owing to possible follow‐up or treatment of patients outside our institution.

## 6. Conclusion

ABO‐incompatible kidney transplantation demonstrated encouraging long‐term outcomes in this cohort, with 91.7% of recipients maintaining a functioning graft after more than 5 years of follow‐up. Our findings suggest that, in the setting of ABO incompatibility, multiple immunological factors may interact and collectively influence the risk of rejection and graft dysfunction, even when their individual effects are not prominent when assessed separately. These findings should be interpreted in light of the retrospective design, limited sample size, and single‐center nature of the study. Nevertheless, they provide exploratory clinical insight and support the need for larger multicenter studies, ideally incorporating standardized immunological assessment and protocol biopsies, to better characterize cumulative immunological risk and refine individualized risk stratification in ABO‐incompatible kidney transplantation.

## Author Contributions

Najla Zabani and Nasser Odah contributed to the study design. Bilal Mohsin and Fatmah Yamani were responsible for writing the paper. Data analysis was conducted by Ms Lama Hefni and Nadeem Shafique Butt. Naief Alhowaiti and Afnan Al Mutairi contributed to the organization of the MS Excel sheet. Bilal Mohsin was involved in writing the abstract, study introduction, discussion, and conclusion. Najla Zabani was the main lead and supervisor of research, with administrative support from Wael Habhab. Proofreading, editing, and correction of mistakes were done by Fatima Yamani and Bilal Mohsin.

## Funding

No funding was received for this manuscript.

## Conflicts of Interest

The authors declare no conflicts of interest.

## Data Availability

The data that support the findings of this study are available from the corresponding author upon reasonable request.
